# Iron Status and Reproduction in US Women: National Health and Nutrition Examination Survey, 1999-2006

**DOI:** 10.1371/journal.pone.0112216

**Published:** 2014-11-06

**Authors:** Elizabeth M. Miller

**Affiliations:** Department of Anthropology, University of South Florida, Tampa, Florida, United States of America; University of Florida, United States of America

## Abstract

Women experience significant changes in iron status throughout their reproductive lifespans. While this is evident in regions with high rates of malnutrition and infectious disease, the extent of reproductive-related changes is less well known in countries with low rates of iron deficiency anemia, such as the United States. The goal of this study is determine the relationship between women's reproductive variables (pregnancy, parity, currently breastfeeding, regular menstruation, hormonal contraceptive use, and age at menarche) and iron status (hemoglobin, ferritin, transferrin receptor, and % transferrin saturation) using an anthropological framework for interpreting the results. Data from women aged 18–49 were taken from the 1999–2006 US NHANES, a nationally representative cross-sectional sample of US women. Using multiple imputation and complex survey statistics, women's reproductive variables were regressed against indicators of iron status. Pregnant women had significantly poorer iron status, by most indicators, than non-pregnant women. All biomarkers demonstrated significantly lower iron levels with increasing parity. Women who were having regular periods had iron indicators that suggested decreased iron levels, while women who used hormonal contraceptives had iron indicators that suggested increased iron levels. Despite relatively good iron status and widespread availability of iron-rich foods in the US, women still exhibit patterns of iron depletion across several reproductive variables of interest. These results contribute to an ecological approach to iron status that seeks to understand variation in iron status, with the hopes that appropriate, population-specific recommendations can be developed to improve women's health.

## Introduction

Globally, reproductively-active women are at risk of iron-deficiency anemia, which causes significant morbidity and mortality [1], [2]. The effects of low iron in women can have broad global effects on their physical and cognitive capabilities as well as specific effects on perinatal outcomes and infant health [3], [4]. During pregnancy, iron is allocated to the fetus to a high degree, particularly in the later trimesters [5], [6]. This can lead to maternal and fetal iron deficiency anemia, particularly in women with poor iron status pre-pregnancy [4], [7]–[9]. There is also evidence that this can affect women across their reproductive careers: a growing body of literature suggests that increasing parity is associated with decreased indicators of iron status and greater likelihood of iron-deficiency anemia [10]–[14]. While pregnancy depletes maternal iron stores, after birth women have relatively low iron needs that allows for repletion of iron stores before the next pregnancy [14]. When inter-birth intervals are short or when dietary iron is insufficient, parity-related maternal iron depletion can result [14], [15].

Iron status has also been implicated in other aspects of women's reproduction, mainly attributed to the loss of iron via menstrual blood. Menstrual blood loss has been associated with poorer indicators of iron status [16]–[18], although this perspective is controversial [19]. Fittingly, the use of hormonal contraceptive, which is generally associated with lighter menstrual periods, is associated with better indicators of iron status than in women who do not use hormonal contraceptives [10], [17]. Breastfeeding is also associated with lower dietary iron needs [20], [21] due to low levels of iron in breast milk and lactational amenorrhea [22]–[25], particularly in undernourished populations.

The National Health and Nutrition Examination Survey (NHANES) offers a unique opportunity to investigate markers of iron status across a broad cross-section of reproductive-aged US women. Compared to the global population, the United States has low rates of iron-deficiency anemia; however, it does appear that reproduction-related iron depletion can occur, particularly in pregnant women [26] and African American women [12]. This study will investigate the relationship between reproductive variables and markers of iron status using a biological anthropology framework. Specifically, it will explore how pregnancy, parity, breastfeeding, menstruation, hormonal contraceptive use, and menarche are associated with four indicators of iron status: hemoglobin, ferritin, transferrin receptor, and percent transferrin saturation. This research will test the following hypotheses:

- Women will show differences in iron status depending on their current and past reproductive history. This hypothesis leads to three predictions: a) Pregnant women will have lower iron status than women who are not pregnant; b) Women who are currently breastfeeding will have iron indicators that indicate post-pregnancy iron recovery; and c) Increasing parity (reproductive history) will be negatively associated with indicators of iron status.
- Women will experience short- and long-term effects of menstruation on iron status. This hypothesis leads to two predictions: a) Regularly menstruating women will have iron indicators that indicate lower iron status; and b) Earlier age at menarche will be associated with lower iron status.

The US NHANES offers an opportunity to examine several indicators of iron status in reproductive-aged women. Hemoglobin, an iron-containing oxygen carrier protein in red blood cells, is the most common iron indicator used to diagnose anemia. Low hemoglobin is diagnostic of anemia (the lowered ability of the blood to carry oxygen) but cannot necessarily distinguish between iron deficiency anemia and other causes of anemia. Serum ferritin, an iron storage protein, correlates well with global iron stores (except in the presence of inflammation). Low serum ferritin is especially useful in distinguishing between iron deficiency anemia and other forms of anemia [27]. Percent transferrin saturation is the percent iron bound to transferrin (an iron carrier protein), and is also a measure of iron deficiency. Finally, serum transferrin receptor binds to transferrin in order to transfer iron into cells. Transferrin receptor increases during iron deficiency as the body's tissues attempt to increase intercellular iron concentration, and can be used to distinguish iron deficiency anemia from other forms of anemia even when inflammation is present. These four measurements offer similar, but slightly different, perspectives on iron status and can provide insight into the dynamics of iron physiology in reproductive-aged women.

## Subjects and Methods

### Ethics statement

This study was originally approved by the National Center for Health Statistics Research Ethics Review Board, and participants underwent informed consent before data collection. Because the current study is a secondary analysis of de-identified data, the University of South Florida Institutional Review Board determined that this research is not human subjects research and thus not subject to review.

### Sample design

The NHANES is a US-representative survey conducted by the Centers for Disease Control and Prevention (CDC), which has been collecting data on a two-year continuous basis since 1999. The goal of the NHANES is to collect health and nutrition-related data on the general US population. NHANES uses a complex sample design in which participants are weighted according to geographic and census information, and certain groups (such as pregnant women) are oversampled for analytical purposes [28]. Adding to the complexity of the data, not all NHANES participants who took part in the interview decided to take part in the physical examination. There are also considerable missing data, particularly in the reproductive health questionnaire. Although 6603 women between the ages of 18 and 49 participated in the physical examination between 1999 and 2006, rates of missing data were fairly high (see Table 1). Therefore, the current study uses a multiple imputation method to correct for missing responses and increase available sample size.

**Table 1:** Weighted descriptive statistics for study variables for women aged 18–49 who participated in the NHANES physical examination during 1999–2006, before and after multiple imputation of missing values.

		Pre-imputed weighted descriptive statistics			Imputed weighted descriptive statistics^a^		
		Mean or %	SE	*n*	Mean or %	SE	95% CI
Iron Status	Hemoglobin (g/dL)	13.50	0.37	6225	13.50	0.036	13.43, 13.57
	Ferritin (ng/mL)	57.84	1.03	6162	57.72	1.05	55.67, 59.78
	% Transferrin saturation	3.57	0.037	2824	3.70	0.042	3.61, 3.78
	Transferrin receptor (mg/L)^b^	22.60	0.19	6153	22.58	1.18	22.22, 22.94
Reproductive Status	% Pregnant	6.43	0.32	6463	6.52	0.32	5.89, 7.16
	Parity	1.54	0.028	5616	1.53	0.027	1.47, 1.58
	% Currently breastfeeding	2.01	0.34	5107	1.90	0.33	1.26, 2.53
	% Currently using hormonal contraceptive	16.02	0.84	5608	15.86	0.78	14.33, 17.39
	% Ever used hormonal contraceptive	77.46	0.86	5942	77.00	0.87	75.28, 78.70
	% Had regular periods in past year	72.40	0.83	5947	72.58	0.84	70.92, 74.23
	Age at menarche (years)	12.60	0.028	5854	12.60	0.027	12.55, 12.65
Covariates	% Hispanic	15.47	0.13	6603	15.47	0.13	–c
	% Non-Hispanic black	13.48	0.11	6603	13.48	0.11	–c
	% Non-Hispanic white	65.45	0.10	6603	65.45	0.10	–c
	% Other	5.60	0.045	6603	5.60	0.045	–c
	BMI (kg/m^2^)	27.86	0.16	6468	27.87	0.16	27.56, 28.19
	CRP (g/L)	0.47	0.014	6173	0.47	0.014	0.44, 0.50
	Age (years)	33.99	0.17	6603	33.99	0.17	–c
	Dietary iron intake (mg)	13.73	0.14	6252	13.73	0.14	13.46, 14.00

a All *n*  =  6603 (except transferrin receptor).

b The *n* for the imputed mean for transferrin receptor is 3295; based on 2003–2006 survey years only.

c All variables were available in original data set, so no 95% CI were generated by PROC MIANALYZE.

### Variable selection

#### Iron status

NHANES 1999–2006 has multiple variables relating to iron status. Information relating to laboratory analysis of iron status variables are available in the NHANES documentation on the CDC website. All continuous variables were left as untransformed linear variables for analysis.

Found in red blood cells, hemoglobin is the main oxygen-transporting protein in the body. Each hemoglobin molecule contains one iron ion. Around 70% of the body's iron is located in hemoglobin. Hemoglobin levels are used to diagnose iron-deficiency anemia, with values of <12–12.5 g/dL generally considered anemic in women [29], although can vary by pregnancy status [3]. In the NHANES, hemoglobin was measured as part of the complete blood count using the Coulter HMX Hematology Analyzer [30]–[33]. Hemoglobin levels (g/dL) are available for all children and adults who completed the physical examination [30]–[33].

Ferritin is an iron-storage protein that is indirectly used as a measure of iron levels in the body. Ferritin levels of <12 ng/mL are considered indicative of iron deficiency [29]. Ferritin levels (ng/mL) are available for all adults and children from survey years 1999–2002 and in reproductive aged-women (12–49 years) in survey years 2003–2006 [30]–[33]. Two different assays were used to measure ferritin across data years: in years 1999–2003 BioRad Laboratories' two-site immunoradiometric assay kit was used, while in 2004 and later years the Roche/Hitachi immunoturbidity assay was used. The Roche/Hitachi method gives a higher ferritin estimate than the BioRad assay, and must be normalized using a derived piecewise linear equation [34]. While the 2003 data were normalized to the 2004 data prior to release, investigators that use the 1999–2002 data with later releases, including the current study, must adjust the earlier values using provided equations [34]. Ferritin levels are increased during acute-phase inflammation [35], so C-reactive protein (CRP) should be included in multivariate models as a control variable.

Transferrin receptor is a carrier protein for transferrin, providing transportation for iron into cells and helping maintain iron homeostasis in the body. Transferrin receptor is upregulated in the case of low body iron in order to help maintain intracellular iron levels, and is frequently elevated in pregnancy [36]–[38]. Previous research has indicated that the cutoff for iron deficiency for transferrin receptor in reproductive-aged women is > 5.33 mg/L [39]. In the NHANES, serum transferrin receptor was measured via immunoturbidity assay using Roche kits on the Hitachi Mod P clinical analyzer [32], [33]. Serum transferrin receptor (mg/L) was available for all women aged 12–49 years in survey years 2003–2006, but was only available for pregnant women in survey years 1999–2002 [30]–[33]. While multiple imputation would theoretically replace the missing data in the earlier survey years, in practice multiple imputation of all 8 years lead to biased data and models that would not converge. Therefore, the decision was made to perform analyses on transferrin receptor for 2003–2006 only, and impute the missing data only in those years.

Percent transferrin saturation is a measure of the total body iron that is bound to transferrin, which is a blood protein that binds to and controls the release of the body's iron. Percent transferrin saturation was calculated using the formula: serum iron/total iron-binding capacity x 100%. Serum iron and total iron-binding capacity were measured using automated AAII-25 colorimetric method modified to be performed on the Alpkem Flow Solutions 3000 system [30]–[33]. Percent transferrin saturation was available in 1999–2000 for men and women of all ages and in 2001–2006 for women between the ages of 12 and 59 years [30]–[33]. Percent transferrin saturation is considered deficient when values fall below 16% [29].

#### Reproductive history variables

Reproduction-related variables are available for all women above the age of 12 in the reproductive health questionnaire. Some reproductive variables were constructed using more than one variable in order to accurately represent the survey methods and the population's response. For all constructed reproductive variables, respondents with missing values for both questions were left missing.

Pregnancy status was determined by the results of the urine pregnancy test. A continuous parity variable was constructed based on a combined variable, first by using a variable that asks if women had ever been pregnant and for those that said yes, using the number of reported live births. Women who had no pregnancies were reported to have a parity of 0; for all others, the reported live births were used as their parity value.

A dichotomous variable for currently breastfeeding women was created using three variables: First, women who reported never being pregnant were coded as not currently breastfeeding. Second, women who had given birth within the past two years were asked if they were currently breastfeeding; those that said yes were coded as currently breastfeeding and those that said no were coded as not currently breastfeeding. Finally, women who had reported giving birth 2 or more years ago were coded as not currently breastfeeding (the NHANES survey made the assumption during data collection that women 2 or more years post birth were not currently breastfeeding).

Two dichotomous variables for hormonal contraceptive use were created: one, for current use of hormonal contraception, and two, for using hormonal contraception at any point during the life span.

Reported having regular menstrual periods over the previous 12 months was included as a dichotomous variable. To assess the long-term effects of menstrual history, recalled age at menarche was included as a continuous variable.

#### Control variables

Regression analyses were controlled for the following variables: Age, CRP level, body mass index (BMI), survey year, ethnicity, dietary iron intake from 24-hour recall, and household income. Age, BMI, CRP, and dietary iron intake were included as continuous variables. Yearly household income was coded as dummy variables in $5000 increments, up to $75,000+. Ethnicity was coded as dummy variables for Hispanic (including Mexican Americans), non-Hispanic black and other, with non-Hispanic white as the reference category. Survey year for each two-year data-release period was included as dummy variables.

Eight-year examination sample weights were calculated using the 4-year weight for survey years 1999–2002 and two-year weights for 2003-2004 and 2005–2006. The 4-year weights were multiplied by ½ and the 2-year weights were multiplied by ¼ to create the 8-year weight variable. To create the 4-year weights for the transferrin receptor models for 2003–2004 and 2005–2006, the 2-year examination sample weights were multiplied by ½ [28].

### Statistical methods

Excluding individuals who have missing values from analysis can lead to biased results [40], [41]. Multiple imputation (MI) is a statistical method for replacing missing variables in a data set. Imputation models use a probability model on both complete and missing data in a set to generate likely variables for missing values. In multiple imputation, several imputed data sets are generated, and the desired statistical analysis is performed on each one. After analysis, the results from each imputed set are averaged across variables to help control for the variance introduced by the imputation process [40], [41].

Multiple imputation proceeds through three steps: 1) the generation of the MI data sets, which generates likely values for missing variables based on available data; 2) complex survey regression analyses based on the MI data sets; and 3) the synthesis of the imputed data sets and regression analyses, which combines the imputed results and reports the variability introduced by the imputation process [41], [42]. The MI process was performed in SAS 9.3 (SAS Institute, Inc., Cary, North Carolina). Statistical significance was assessed at α = 0.05 (two-sided).

The multiple imputation of data sets was performed using PROC MI in SAS. In SAS, PROC MI uses a Markov chain Monte Carlo method that assumes arbitrary missing data and multivariate normality. Standard usage of MI data sets suggests that at least 5 imputed data sets should be used, although this number may be higher for data sets with more missing data [43]. Due to the high levels of missing values for some variables in the data set, *n* = 50 imputations were performed in this analysis. For 50 imputations, reliability estimates for each variable were above 95%. All variables mentioned above were included in the imputation analysis, but income variables were dropped from regression analysis because they were not statistically significant and had no biological rationale for being included as a control variable.

A special mention should be made of dichotomous variables in MI procedures. Imputed dichotomous variables are not dichotomous themselves, but instead range as a proportion between 0 and 1. Some practitioners round the imputed fractions to the nearest 0 or 1; however, this practice leads to biased estimates of these variables [44]. Analysis of a variety of methods for handling dichotomous variables suggests that for the majority of cases, imputed fractions should be left alone for regression analysis [45]. Therefore, imputed dichotomous variables in this study were left as-is for analysis.

Analysis of imputed data sets was performed using PROC SURVEYMEANS for descriptive statistics and PROC SURVEYREG for linear regression. All survey analyses (descriptive and regression) were adjusted using NHANES-provided variables for strata and cluster, and the adjusted 8-year sample weight described above (or the 4-year sample weight for the transferrin receptor model). The imputed data sets were added to the model as part of the domain statement. This allows the analyses to be performed on each imputed data set.

For the PROC SURVEYREG analyses, four models were run with each iron biomarker (hemoglobin, ferritin, transferrin receptor, and % transferring saturation) as dependent variables. Independent variables for each model were as follows: current pregnancy, parity, currently breastfeeding, currently using contraceptive pills, ever used contraceptive pills, having regular periods in the past 12 months, and age at menarche. All models were adjusted for ethnicity (with white ethnicity as the reference variable), survey release years (with 1999–2000 as the reference variable except the transferrin receptor model, which used 2003–2004 as the reference variable), BMI, CRP, age, and 24-hour recall of dietary iron intake.

To complement MI analysis, it is recommended that an analysis of all complete cases (cases with no missing data) be performed in order to assess potential areas of bias, either in the complete case or in the multiple imputation [46]. In this study, complete case analyses were performed for each model using PROC SURVEYREG and was adjusted for examination weight, strata, and cluster as described above. The subdomain for this analysis was female exam participants ages 18–49. Descriptive statistics for the non-imputed, original data were performed using PROC SURVEYMEANS and the parameters described above.

The final step was performed using PROC MIANALYSIS. This procedure synthesizes the results of the 50 imputed data sets, providing summary means and adjusted variances for PROC SURVEYMEANS, and summary parameter estimates and adjusted variances for the results of PROCSURVEYREG. This method also provides 95% confidence intervals for means and parameter estimates. Using these three steps, results are adjusted for both the multiple imputation process and the complex survey design of NHANES.

## Results

**Figure 1:**
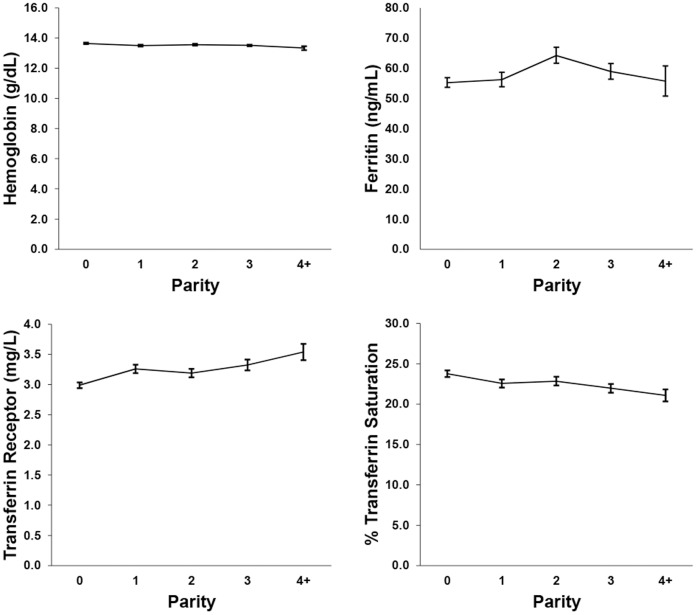
Weighted (unimputed) means and ±1 standard error of the mean for measures of iron status by pregnancy status.

**Figure 2:**
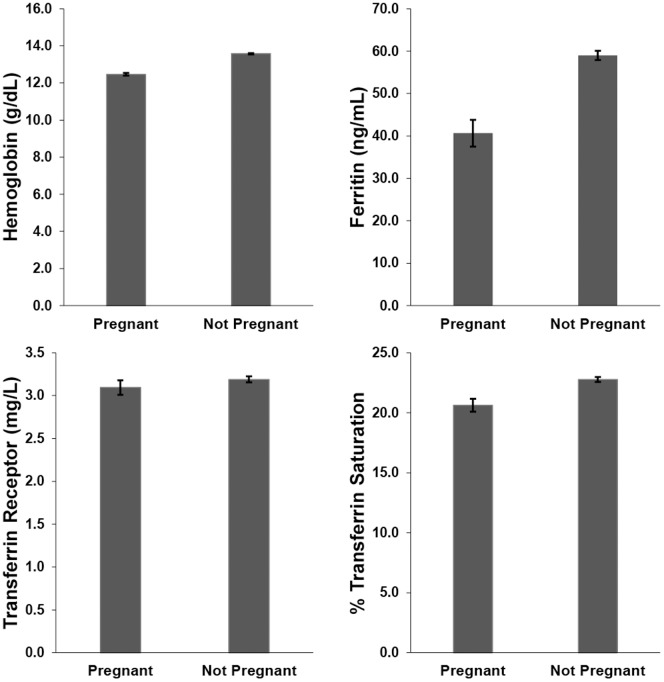
Weighted (unimputed) means and ±1 standard error of the mean for measures of iron status by parity.

Descriptive statistics were performed on both the original data sets and the imputed data sets using PROC SURVEYMEANS, to adjust for the complex survey design (Table 1). The percent missing data, derived from the total number of eligible women (*n* = 6603), ranged from 0% to 32.7% depending on the variable of interest. Graphs of the weighted association between iron biomarkers and pregnancy are found in Figure 1 and iron biomarkers and parity in Figure 2. Hemoglobin, ferritin, and % transferrin saturation declined with increasing parity and was also reduced in pregnant women. Transferrin receptor increased with increasing parity, and was higher in non-pregnant women compared to pregnant women. Table 2 shows the percentage of women who fall below the cutoff value for each iron biomarker, as well as the percentage of women considered iron deficient, as defined by having two out of three values of hemoglobin, ferritin, and % transferrin saturation below their respective cutoff values [47].

**Table 2:** Non-imputed, weighted percentages of NHANES women who are below iron indicator cutoffs by pregnancy status (See Table 1 for number of non-missing values in each category).

	Non-pregnant women	Pregnant women
Hemoglobin (<12 g/dL)	6.9%	29.1%
Ferritin (<12 ng/mL)	10.9%	18.5%
Transferrin receptor (>5.33 mg/L)^a^	5.4%	5.7%
% Transferrin saturation (<16%)	29.7%	39.1%
% Iron deficient^b^	9.8%	25.4%

a Percentages based on 2003–2006 survey years only.

b Calculated based on percentage of women who had two of three values (hemoglobin, ferritin, and % transferrin saturation) below cutoff.

Complete case results (estimates and p-values) for survey regression for the four models is found in Table 3. In general, there were between 60–65% complete cases for each model out of the eligible women in the study population. Results do not appear to differ significantly between the complete case results and the imputed results, reported in Table 4.

**Table 3:** Weighted estimates and p-values for complete case survey regression models of hemoglobin, ferritin, % transferrin saturation, and transferrin receptor as dependent variables, reproductive variables as independent variables, and ethnicity, survey release year, and other covariates as control variables.

		Hemoglobin		Ferritin		% Transferrin Saturation		Transferrin Receptor^a^	
		*n* = 4255		*n* = 4252		*n* = 4245		*n* = 2019	
		R^2^ = 0.15		R^2^ = 0.071		R^2^ = 0.095		R^2^ = 0.099	
		Estimate	*p*	Estimate	*p*	Estimate	*p*	Estimate	*p*
Reproductive Status	Currently pregnant	−1.02	<0.0001	−19.94	<0.0001	−0.58	0.48	−0.033	0.74
	Parity	−0.081	0.0003	−2.27	0.0026	−0.66	0.0002	0.047	0.17
	Currently breastfeeding	0.037	0.78	3.41	0.5687	−0.98	0.35	0.37	0.14
	Current birth control use	−0.034	0.59	9.97	0.0108	1.44	0.042	−0.33	0.0001
	Ever use birth control	0.026	0.69	−0.062	0.98	−0.042	0.95	−0.00070	0.99
	Having regular periods	−0.20	0.0032	−16.62	<0.0001	−1.22	0.067	0.013	0.93
	Age at menarche	−0.024	0.076	−0.65	0.3056	−0.070	0.59	−0.0051	0.74
Ethnicity^b^	Hispanic	−0.29	<0.0001	−4.09	0.0355	−1.49	0.016	−0.053	0.60
	Non-Hispanic black	−1.06	<0.0001	−0.022	0.9935	−2.84	<0.0001	0.54	<0.0001
	Other	−0.47	0.0004	7.73	0.0711	−1.85	0.034	0.29	0.12
Survey Release^c^^,^ d	2001–2002	−0.033	0.70	−10.39	0.0011	−0.22	0.75	–	–
	2003–2004	0.14	0.11	2.61	0.4303	0.41	0.60	–	–
	2005–2006	0.094	0.28	0.14	0.9627	0.025	0.97	−0.25	0.0023
Other Covariates	BMI (kg/m^2^)	0.0018	0.55	0.11	0.5819	−0.27	<0.0001	0.029	0.0002
	CRP (g/L)	−0.087	0.0017	10.54	<0.0001	−2.34	<0.0001	0.053	0.18
	Age (years)	−0.00020	0.96	0.73	<0.0001	0.040	0.16	−0.00060	0.90
	Dietary iron intake (mg)	−0.00010	0.95	0.044	0.7448	−0.013	0.63	0.0043	0.32

a Transferrin receptor model is based on survey years 2003–2006 only.

b Reference category is white ethnicity.

c Reference category is survey release year 1999–2000 (hemoglobin, ferritin, and % transferrin saturation models).

d Reference category is survey release year 2003–2004 (transferrin receptor model only).

**Table 4:** Weighted estimates, 95% confidence intervals, and p-values for imputed survey regression models of hemoglobin, ferritin, % transferrin saturation, and transferrin receptor as dependent variables, reproductive variables as independent variables, and ethnicity, survey release year, and other covariates as control variables.

		Hemoglobin^a^			Ferritin^a^			% Transferrin Saturation^a^			Transferrin Receptor^b^		
		R^2^ = 0.17^c^			R^2^ = 0.12^c^			R^2^ = 0.093^c^			R^2^ = 0.10^c^		
		Estimate	95% CI	*p*	Estimate	95% CI	*p*	Estimate	95% CI	*p*	Estimate	95% CI	*p*
Reproductive Status	Currently pregnant	−1.12	−1.24, −0.99	<0.0001	−19.60	−27.03, −12.16	<0.0001	−0.58	−1.84, 0.67	0.36	−0.29	−0.51, −0.057	0.014
	Parity	−0.066	−0.10, −0.030	0.0003	−2.94	−4.55, −1.34	0.0003	−0.62	−0.90, −0.34	<0.0001	0.10	0.052, 0.15	<0.0001
	Currently breastfeeding	−0.076	−0.34, 0.19	0.57	−7.40	−21.12, 6.32	0.29	−0.95	−3.38, 1.48	0.44	0.37	−0.14, 0.88	0.15
	Current birth control use	−0.077	−0.20, 0.043	0.21	14.74	6.24, 23.23	0.0007	1.18	−0.084, 2.45	0.067	−0.40	−0.57, −0.23	<0.0001
	Ever use birth control	0.057	−0.056, 0.17	0.32	0.56	−4.36, 5.47	0.82	−0.0058	−1.08, 1.06	0.99	−0.059	−0.25, 0.13	0.55
	Having regular periods	−0.28	−0.37, −0.19	<0.0001	−32.25	−38.43, –26.08	<0.0001	−1.42	−2.34, −0.50	0.0025	0.29	0.068, 0.52	0.011
	Age at menarche	−0.012	−0.035, 0.011	0.29	−0.70	−1.91, 0.51	0.26	0.022	−0.20, 0.25	0.84	0.015	−0.024, 0.053	0.45
Ethnicity^d^	Hispanic	−0.25	−0.35, −0.14	<0.0001	−4.09	−7.92, −0.26	0.036	−1.59	−2.55, −0.63	0.0012	0.042	−0.13, 0.21	0.62
	Non-Hispanic black	−1.08	−1.17, −0.98	<0.0001	6.57	−0.27, 13.41	0.060	−2.53	−3.34, −1.73	<0.0001	0.91	0.68, 1.14	<0.0001
	Other	−0.31	−0.49, −0.13	0.0008	11.76	2.37, 21.16	0.014	−1.45	−2.89, −0.0058	0.049	0.086	−0.15, 0.33	0.48
Survey Release^e^^,^ f	2001–2002	−0.0031	−0.18, 0.18	0.97	−6.80	−12.06, −1.53	0.012	−0.10	−1.17, 0.96	0.85	—	—	—
	2003–2004	0.21	0.057, 0.37	0.0077	9.38	4.95, 13.81	<0.0001	0.94	−0.20, 2.08	0.11	–	–	–
	2005–2006	0.11	−0.05, 0.28	0.17	3.11	−2.18, 8.40	0.25	0.054	−1.00, 1.11	0.92	−0.052	−0.21, 0.10	0.51
Other Covariates	BMI (kg/m^2^)	0.00064	−0.0043, 0.0056	0.80	0.23	−0.17, 0.62	0.26	−0.27	−0.31, −0.22	<0.0001	0.033	0.025, 0.042	<0.0001
	CRP (g/L)	−0.032	−0.082, 0.017	0.20	12.61	7.49, 17.73	<0.0001	−1.96	−2.43, −1.48	<0.0001	−0.016	−0.090, 0.057	0.66
	Age (years)	0.000067	−0.0050, 0.0052	0.98	1.16	0.90, 1.41	<0.0001	0.067	0.021, 0.11	0.0046	−0.0026	−0.012, 0.0065	0.58
	Dietary iron intake (mg)	−0.0017	−0.0062, 0.0028	0.45	−0.12	−0.37, 0.14	0.37	−0.024	−0.067, 0.019	0.27	0.0024	−0.0063, 0.011	0.59

a The *n* for the hemoglobin, ferritin, and % transferrin saturation model is 6603 (all years).

b The *n* for the transferrin receptor model is 3295 (survey years 2003–2006 only).

c Mean R^2^ of 50 imputations.

d Reference category is white ethnicity.

e Reference category is survey release year 1999–2000 (hemoglobin, ferritin, and % transferrin saturation models).

f Reference category is survey release year 2003–2004 (transferrin receptor model only).

Imputed estimates, 95% confidence intervals, and p-values for each of the four models (analyzed using imputed values and PROC SURVEYREG) can be found in Table 4. In the hemoglobin model, there was a significant negative association between pregnancy and hemoglobin, parity and hemoglobin, and having regular periods and hemoglobin. In addition, white American women had significantly higher hemoglobin than all other ethnicities.

In the ferritin model, ferritin was significantly negatively associated with pregnancy, parity, and having regular periods. Ferritin was significantly positively associated with taking hormonal contraceptive. Several covariates were also statistically significant. Hispanic women had significantly lower ferritin levels while women whose ethnicity was given as “other” had significantly higher ferritin levels. Ferritin was also significantly positively associated with current age and CRP level.

Transferrin receptor levels were significantly positively associated with having regular menstrual periods and parity. Transferrin receptor was negatively associated with pregnancy and taking hormonal contraceptive pills. Transferrin receptor levels were also significantly higher in non-Hispanic black women and in women with higher BMIs.

Percent transferrin saturation was significantly negatively associated with parity and having regular menstrual periods. Hispanic and non-Hispanic black women had significantly lower % transferrin saturation than white women. Finally, % transferrin saturation was significantly negatively associated with BMI and CRP.

## Discussion

Pregnancy had a clear effect on some, but not all, of the iron status measures. Pregnancy was associated with lower levels of hemoglobin and ferritin, indicating that iron availability to red blood cells and iron storage is compromised during pregnancy. This is a typical finding for pregnant women, as the fetus's high iron needs depletes mothers' iron stores, particularly as pregnancy progresses. Similarly, % transferrin saturation was lower in pregnant women than non-pregnant women, but not significantly so. Curiously, transferrin receptor levels were significantly lower in pregnant women compared to non-pregnant women. As transferrin receptor is usually higher under conditions of low body iron, this result is opposite of what would be predicted by previous literature [36], [37]. However, it may be an expected result in the context of maternal-fetal iron transfer. Maternal physiology is hypothesized to negotiate the allocation of resources between maternal and fetal somatic needs, which may sometimes conflict with fetal interests [48], [49]. In the case of maternal-fetal iron transfer, previous research suggests that fetal iron transfer has priority over maintaining maternal iron stores [50], and that fetal iron deficiency only occurs after maternal iron stores have been severely compromised [51], [52]. The current results suggest that transferrin receptor may be downregulated during pregnancy in an attempt to allocate iron away from the mother's tissues and toward the fetus. These results hint at a pregnancy iron transfer system that depletes bodily iron during pregnancy in favor of fetal iron stores, supporting previous findings. However, more research is necessary to confirm the physiological mechanisms that may underlie such a mechanism.

There were no significant effects of breastfeeding on any iron status values. Unfortunately, analysis of this variable was hampered by the low rate of breastfeeding in US women: only 2% of women reported that they were currently breastfeeding. Replicating these findings in a population of women with higher breastfeeding rates would better test the hypothesis that the postpartum period is a time of iron repletion in reproducing women.

These results provide evidence that postpartum iron repletion is incomplete in US women, and has an additive negative effect with each child. Increasing parity was found to have a small but statistically significant impact on all indicators of iron status. These results replicate parity-related maternal iron depletion findings in developing countries with high rates of iron-deficiency anemia, albeit with smaller statistical estimates [14]. Despite the relatively good overall iron status of the US population, high-parity women are vulnerable to poor iron status. This effect may be particularly worrisome in high parity women who become pregnant [26].

The results show that having regular periods across the past 12 months is associated with lower iron stores than not having regular periods, and that taking hormonal contraceptive pills is associated with higher iron status. This seemingly points to the traditional view that menstrual blood loss directly affects iron status, and that contraception's protective effect is due to lighter menstrual periods while on the pill [16], [17]. More recently, however, mouse models have demonstrated a direct relationship between estrogen and iron homeostasis [53], [54]. Work on this relationship shows that that estrogen directly inhibits the expression of hepcidin, a liver-produced peptide hormone that inhibits iron intake across the gut and is a regulator of iron homeostasis in the body [55]. When estrogen is high, hepcidin is low and iron uptake into the body is increased. The authors proposed that this is a mechanism to regulate iron uptake across the menstrual cycle, and can explain the higher level of iron in women who take hormonal contraceptives [55].

Despite the immediate impact of having regular periods and using contraceptive pills on iron status, there appears to be no long-term effects: history of contraceptive pill use and age at menarche were not significantly associated with iron stores in this population. It could be hypothesized that menstruation-related iron loss should accumulate, particularly in a population who spends a high proportion of their reproductive careers menstruating [56]. However, these results call into question the idea that blood loss is the sole cause of altered iron stores in menstruating women. This perspective has also been advanced by Clancy et al. [19], who note that a thicker endometrium (and greater potential menstrual blood loss) is actually associated with higher hemoglobin in reproductive-aged Polish women. Contrary to established wisdom, their work shows menstruation is associated with better, not worse, iron status, and suggests that iron is a sensitive indicator of reproductive condition. Rather than assume that menstrual blood loss leads to anemia, a closer examination of the co-relationship between hepcidin, iron absorption, estrogen, and reproduction in women is warranted. It is more likely that menstruation-related iron homeostasis is tightly regulated, even in women who continually menstruate throughout their reproductive career.

The results showed significantly different measures of iron status between ethnic groups in the US. Non-Hispanic white women had higher hemoglobin compared to other groups, Hispanic women had lower ferritin and % transferrin saturation compared to the reference group (non-Hispanic white women), and non-Hispanic black women had higher levels of transferrin receptor and lower % transferrin saturation compared to the reference group. These differences raise several questions. First, what is the normal range of variation in US women? Why does it vary between groups, and what are the factors that contribute to this reaction norm? Some researchers suggest a lower threshold for iron-deficiency anemia for African-American women [3], for example, but what drives this difference? Second, it also demonstrates that there may be no one picture of low iron status in women, and that each iron indicator may offer a slightly different interpretation of the physiological processes involved in the body's response to low iron. Further research would untangle the meaning of these different pathways, particularly in the context of women's reproduction. Finally, it is worth investigating population-specific reproductive outcomes due to poor iron status. There are well-known consequences of maternal iron-deficiency anemia, including preterm birth, low birth weight, increased maternal morbidity, increased risk of infant iron-deficiency, poor neurocognitive development in infants, and others [4], [7]. However, meta-analyses indicate that most of these adverse outcomes (with the exception of pre-term birth) are not consistent across studies [57]. Rather than doubt the possibility of these adverse outcomes, an anthropological approach would instead posit that there may be ecological variation in the appearance of these outcomes. Instead, the question becomes: who do these adverse outcomes happen to, and why? Further work from an anthropological perspective may provide better insight into the ecology of iron use in women's reproduction.

One interesting finding in this study is that reported levels of dietary iron intake in the current study were not significantly associated with indicators of iron status in statistical analyses. Interestingly, the reported dietary intake of iron in this sample of women (13.73 mg/day) was lower than the recommended daily intake of 18 mg/day for reproductive-aged women; however, rates of iron deficiency were 9.8% in non-pregnant women and 25.4% in pregnant women (Table 2). These results show that while women in the US have clear reproductive-related changes in iron status, low iron is considerably less prevalent in non-pregnant women, despite their lower-than-recommended dietary intake.

When viewed through an anthropological framework, these results challenge current interpretations of the variation in women's reproduction in several ways. First, rather than use cut-off levels that determine iron deficiency, this study instead examined iron status as continuous variables, as is typical in biological anthropology. Therefore, these results do not make recommendations relating to supplementation or the avoidance of reproductive-related low iron in the US. Rather, they are intended to show associated patterns and to help identify potential future research areas of interest to both anthropologists and nutritional scientists. Second, although many of these results are statistically significant, they may not all be biologically significant. For example, the parity results, although significant, would require a large number of children in most cases to find a biologically meaningful effect. While this is uncommon among US women, populations with higher fertility rates should be advised of parity-related effects. Third, the results from this study challenge what is considered a “normal” iron status in US women. These results show evidence of reproduction-related changes in iron status in US women despite the widespread availability of iron-rich foods and supplements. Perhaps rather than viewing every case of low iron during pregnancy as a problem in need of correction, low iron should be viewed as a normal function of women's pregnancy, provided these women and their infants do not experience adverse outcomes [58], [59]. This falls in line with other research that suggests that pregnant women should have lower cutoff thresholds for anemia, and that these cutoffs may vary by ethnicity [3]. Finally, these results highlight the contradictory nature of recommending supplementation while stating that some degree of low iron is normal in reproducing women. To reconcile the contradiction, it may be true that US women need iron supplementation during their pregnancy, but might not need the daily high doses of iron recommended by health officials. For example, a meta-analysis of the literature has found that intermittent iron supplementation prevents iron deficiency in pregnant women as well as daily supplementation, with fewer adverse effects [60]. The current results do suggest that certain situations may require more attention to risk factors that might require iron supplementation, such as very high parity women and non-white women. By incorporating some tolerance of low iron as “normal,” and by understanding the ecological variation in iron status between populations, supplementation recommendations can help avoid under- and over-treating low iron in reproductive-aged women. These results can help point researchers to more specific iron supplementation recommendations for pregnant and non-pregnant women, both in the US and on the global stage.

There are several limitations to this study. First, these results are limited by the data collection. The NHANES was not specifically designed for a study of this nature, so data are limited and missing in many cases. This was partially corrected by means of multiple imputation, but the limitations on data between survey releases could not be statistically overcome, particularly in the case of transferrin receptor data. Similarly, there are limited types of questions available in the survey, and not all questions of interest could be asked using this data. For example, women's interbirth interval is a very important data point when considering the pregnancy-depletion/postpartum-repletion cycle and parity-related iron depletion. Despite these limitations, these results offer insight into the mechanisms of reproductive-related iron status in US women and suggest future research into the mechanisms of reproductive iron homeostasis.
